# Protein Structural Modeling Explains Rapid Oxidation in Poultry and Fish Myoglobins Compared to Livestock Myoglobins

**DOI:** 10.3390/proteomes13040050

**Published:** 2025-10-08

**Authors:** Greeshma Sreejesh, Surendranath P. Suman, Gretchen G. Mafi, Morgan M. Pfeiffer, Ranjith Ramanathan

**Affiliations:** 1 Department of Animal and Food Sciences, Oklahoma State University, Stillwater, OK 74075, USA; greeshma.sreejesh@okstate.edu (G.S.); gretchen.mafi@okstate.edu (G.G.M.); morgan.pfeiffer@okstate.edu (M.M.P.); 2 Department of Animal and Food Sciences, University of Kentucky, Lexington, KY 40546, USA; spsuma2@uky.edu

**Keywords:** myoglobin, oxidation, solvent-accessible surface area, cavity space, bioinformatics, protein modeling

## Abstract

Background: This study aimed to investigate rapid oxidation in poultry and fish myoglobin compared to livestock myoglobin using protein structural differences and bioinformatics tools. Methods: Myoglobins from beef (*Bos taurus*), bison (*Bos bison*), sheep (*Ovis aries*), goat (*Capra hircus*), red deer (*Cervus elaphus*), pork (*Sus scrofa*), chicken (*Gallus gallus*), turkey (*Meleagris gallopavo*), yellowfin tuna (*Thunnus albacares*), and tilapia (*Oreochromis niloticus*) were analyzed to understand differences in structure and function that may influence oxidative behavior. Results: Fish and poultry had shorter or absent D-helix in their myoglobin structure than other species. Tilapia showed the largest heme cavity surface area, indicating significant internal void space, while yellowfin tuna had the largest heme cavity volume, which could affect ligand binding dynamics compared with poultry and other livestock species. However, the heme solvent-accessible area was greater in chicken and turkey than in fish and other livestock species. Tuna myoglobin contains a cysteine and fish myoglobins have fewer amino acids compared to other species. Limited knowledge is currently available on the effects of proteoform, especially post-translational modifications, on the oxidation of myoglobin from different species. Conclusions: The bioinformatics approach used in this study suggests that, in addition to physiological reasons, shorter D-helix, larger heme cavity in tilapia and yellowfin tuna, and greater solvent-accessible area in poultry contribute to increased oxidation in myoglobin from poultry and fish compared with myoglobin from livestock species.

## 1. Introduction

**Figure 1:**
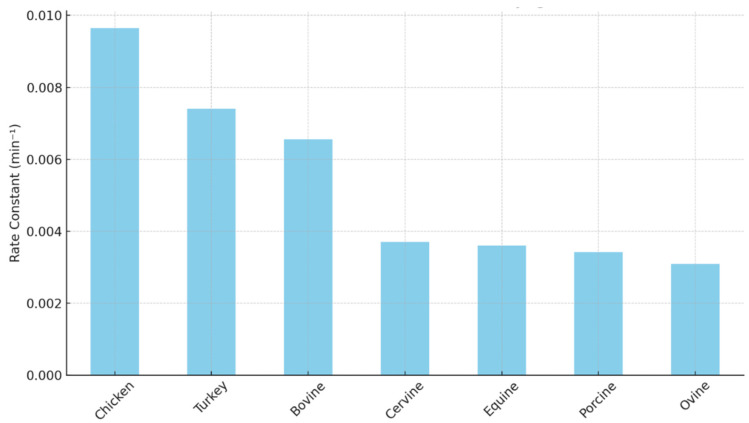
Oxymyoglobin oxidation rate in different species. The first-order rate constant was calculated based on oxymyoglobin oxidation data from Yin et al. 2011 [4]. Tuna myoglobin oxidizes 2.5 to 3 times faster than equine myoglobin [6].

Meat color is a key sensory attribute that affects quality perception and purchasing decisions. Of the several heme proteins, myoglobin is the primary contributor to meat color. Physiologically, the role of myoglobin is to transport oxygen and serve as an oxygen depot for cellular activity. However, after the animal is harvested, its metabolism changes from aerobic to anaerobic. Myoglobin remains capable of binding to oxygen in postmortem muscle, contributing to the color of meat. Depending on the iron oxidation state and the ligand attached, myoglobin can exist in oxymyoglobin (oxygen ligand and Fe^2+^), deoxymyoglobin (no ligand and Fe^2+^), and metmyoglobin (water is the ligand and Fe^3+^) [1]. The concentration of myoglobin differs between species; hence, the hue and saturation of meat vary widely. The main reason for differences in concentration is variation in fiber type, which results from evolutionary and functional implications [2]. For example, beef has more slow-twitch red fibers, while pork has more fast-twitch white fibers, which are low in myoglobin [2]. Even within an animal, depending on the physical activity and anatomical location, myoglobin concentration varies, hence the meat color and its stability [3]. A previous study on oxymyoglobin oxidation of eight different species (Figure 1) concluded that chicken myoglobin oxidizes faster compared with seven other species [4]. Furthermore, bovine myoglobin oxidized faster than porcine myoglobin. Equine myoglobin (often used as a model myoglobin to study oxidation) had a similar oxidation rate to porcine myoglobin [4]. In a study comparing the autoxidation rate of bovine, ovine, porcine, and cervine oxymyoglobin, it was noted that porcine myoglobin had a lower oxidation rate than the other species [5]. Bluefin tuna myoglobin oxidized faster than equine myoglobin at pH 5.6 and 6.4 [6]. Thus, there are inherent differences in myoglobin oxidation rates among different species.

The 4-hydroxy-2-nonenal (HNE) is a secondary lipid oxidation product formed from oxidation of omega-6 polyunsaturated fatty acids such as linoleic acid and arachidonic acid. HNE is often used as an indicator of lipid oxidation and as a model compound to examine the interaction between lipid and myoglobin oxidation [7]. The research determining the role of HNE in oxymyoglobin oxidation noted that the number of histidines plays an important role in the susceptibility of HNE-induced oxidation [4]. Hence, species with a greater number of histidine residues oxidize faster in the presence of HNE than those with fewer histidine residues. Among the various amino acid residues within myoglobin, the roles of proximal (histidine 93) and distal histidine (64) residues have been studied in detail. For example, proximal histidine binds to heme, while the distal histidine interacts and stabilizes ligands such as oxygen or carbon monoxide. Stabilizing the binding of distal histidine is vital to minimize myoglobin oxidation [8]. Similarly, weaker hydrogen bonding with distal histidine also increases oxidation. The role of distal histidine in minimizing heme oxidation was verified by mutating amino acids around the heme cavity [9]. A stronger hydrogen bond with distal histidine leads to less heme oxidation. Furthermore, a lower pH favors the protonation of the distal histidine and increases heme oxidation.

Both proximal and distal histidine also modulate oxygen binding to heme. Proximal histidine and its surrounding amino acids can influence the hydrogen-bonding network, which modulates the histidine’s basicity and thereby alters its electron-donating capacity to the iron center [10]. Increased electron donation from amino acids stabilizes the ferrous state but weakens the Fe–O_2_ bond, resulting in decreased oxygen affinity. Additionally, the rotational orientation of the proximal histidine imidazole ring (e.g., staggered versus eclipsed) influences the Fe–His bond geometry, with the staggered conformation generally favoring stronger O_2_ binding [10]. The Fe–His bond distance is also critical; shorter distances tend to stabilize O_2_ binding, while longer distances facilitate ligand dissociation. On the distal side, hydrogen bonding between the bound O_2_ and distal residues (e.g., His64, Tyr, Gln) enhances ligand stabilization and reduces dissociation rates, thereby increasing affinity. The electrostatic potential of the distal pocket further modulates binding, with negatively charged or polar environments favoring oxygen retention. Further, steric constraints within the distal pocket affect ligand accessibility and stabilization, where more confined pockets can either hinder O_2_ entry or reinforce ligand retention [11].

Computational protein modeling has emerged as a powerful and indispensable tool to predict the three-dimensional (3D) structure of proteins from their amino acid sequence. The present study utilizes homology modeling, a key bioinformatic technique, for predicting protein structures. Homology modeling, also known as comparative modeling, predicts the 3D structure of proteins when an experimentally determined structure of a related protein (template) is available [12,13]. The approach is based on the general observation that proteins with similar sequences have similar structures. The accuracy of the model depends on the availability and quality of the template structure. Furthermore, the accuracy of the model also depends on the sequence identity; below 30% sequence identity, the quality of the model drops significantly [13,14]. In the absence of experimental structures, computational models can provide valuable insight into a protein’s function. Especially, the X-ray crystal structure of myoglobin from most livestock, avian, and aquatic species is not available. Hence, a computational protein model is a valuable tool for understanding species-specific myoglobin oxidation.

Although it is known that the structure of myoglobin can influence its function, limited studies have utilized myoglobin structure to study why chicken and fish myoglobins oxidize faster than those of other livestock species. The objective of this study was to use a bioinformatics approach to examine how myoglobin sequences and structural properties can aid in identifying greater myoglobin oxidation in poultry and fish compared to ruminants and non-ruminants. This research considered ten species that are good sources of muscle foods.

## 2. Materials and Methods

**Figure 2:**
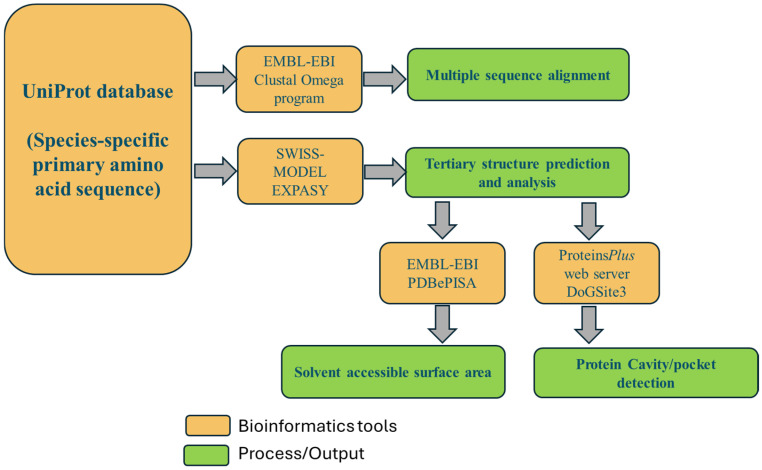
Bioinformatics tools utilized in this research.

Several bioinformatics tools were employed in the study. Figure 2 illustrates the bioinformatics workflow for characterizing the structural and functional properties of myoglobin. These bioinformatics tools were used to provide a comprehensive structural and functional evaluation across multiple vertebrate species.

Myoglobin amino acid sequences of 10 species were retrieved from the UniProt database [15], using the following accession numbers: beef (P02192; *Bos taurus*), bison (P86873; *Bos bison*), sheep (P02190; *Ovis aries*), goat (B7U9B5; *Capra hircus*), red deer (P02191; *Cervus elaphus*), pork (P02189; *Sus scrofa*), chicken (P02197; *Gallus gallus*), turkey (G1NJB6; *Meleagris gallopavo*), yellowfin tuna (P02205; *Thunnus albacares*), and tilapia (I3KNL0; *Oreochromis niloticus*). Table 1 shows the category of species and its amino acid sequence. To represent the biologically relevant mature protein, the N-terminal methionine was excluded from the sequence before modeling and is accordingly absent from the sequences used for this study.

Multiple sequence alignment (MSA) was performed using Clustal Omega with default settings [16]. A percentage identity matrix was generated to evaluate pairwise sequence conservation, and results were visualized as a heatmap using Seaborn (version 0.11.12) in Python (version 3.9.7). The alignment revealed high conservation among phylogenetically related species, such as chicken and turkey, while greater divergence was observed between mammalian and fish myoglobins.

STRIDE is a knowledge-based secondary structure identification algorithm that utilizes atomic coordinates of proteins, hydrogen bonds, and dihedral angles to identify secondary structural elements of proteins [17]. The different types of secondary structures were recognized by using the STRIDE program web interface. Each modeled protein structure from various species was given as input data in PDB file format to compute and visualize the types of secondary structures in the modeled proteins. Different α-helices of myoglobin protein, such as A-H, were identified in the protein structure of various species. The 4th α-helix and D-helix in all species were analyzed and compared to understand the species-specific differences.

The three-dimensional structures of myoglobin proteins from ten vertebrate species were predicted using the SWISS-MODEL homology modeling web-server [12]. Input data included amino acid sequences in FASTA format. The query sequences were aligned to potential structural templates using BLAST [18] and HHblits3 [19] against the SWISS-MODEL Template Library (SMTL). Templates were selected based on evolutionary similarity and ranked by Global Model Quality Estimate (GMQE) scores. The top-ranked models were constructed and retrieved in PDB format for further structural analysis.

The availability of experimentally determined myoglobin structures in public databases is limited. Hence, the validation of the modeled protein structure was done by comparing the protein model with the pork myoglobin X-ray crystal structure 1MWD and the available AlphaFold structure. The beef and pork species share 88% sequence identity (exactly same amino acids) and 92% sequence similarity (evolutionarily substituted amino acids with similar biochemical properties). The modeled beef myoglobin structure was also compared with an AlphaFold predicted bovine structure AF-P86873 that had 99% similarity and identical sequences. Model quality was assessed using established metrics: LDDT (Local Distance Difference Test) [20], TM-score (Template Modeling Score) [21], QMEAN (Qualitative Model Energy Analysis) [22], MolProbity [23], and RMSD (Root Mean Square Deviation). A comparative analysis was performed by superimposing the structures. It was observed that our modeled protein structures were well aligned with the experimental and AlphaFold predicted structures. This supports the reliability of our protein models.

Predicted structures were visualized using PyMOL 3.0 [24] to examine residue-level interactions and heme pocket orientation. The models were then submitted to the ProteinsPlus webserver, which hosts multiple structure-based analysis tools. Pocket detection and characterization were performed using DoGSite3, a grid-based algorithm that quantifies geometric and physicochemical features of cavities. Outputs included pocket volume, surface area, element descriptors, and functional group profiles [25].

To assess solvent accessibility, the predicted structures were also analyzed using the PDBePISA v1.52 (Protein Interfaces, Surfaces and Assemblies) tool provided by EMBL-EBI [26]. This tool calculates the solvent-accessible surface area (SASA), distinguishing between accessible, buried, and interfacial regions within the protein structure. SASA data were extracted for each species-specific myoglobin to facilitate comparison of heme exposure and its potential role in oxidative susceptibility.

## 3. Results

### 3.1. Comparative Study of Myoglobin Primary Sequence Analysis from Multiple Species

**Figure 3:**
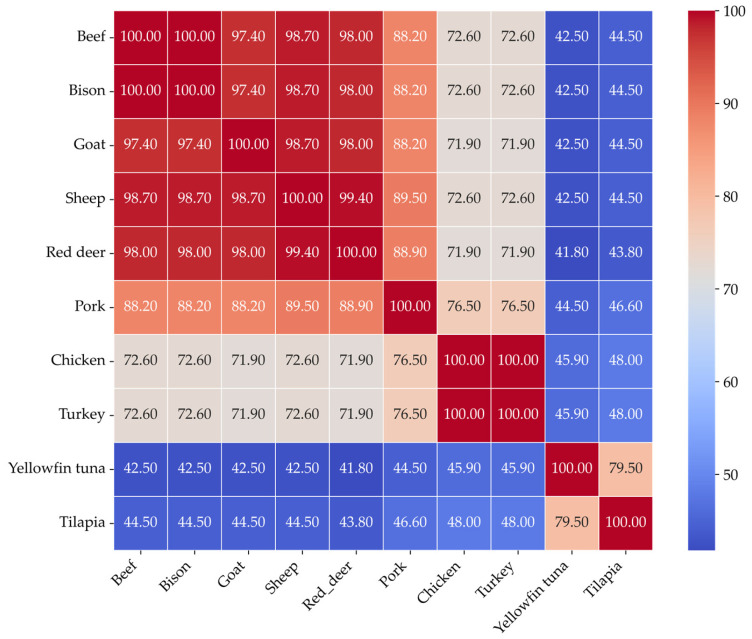
Myoglobin sequence identity heatmap showing pairwise sequence similarity (%) calculated from multiple sequence alignment using Clustal Omega.

Multiple sequence alignment of myoglobin proteins from ten different species showed distinct patterns of sequence conservation. These patterns directly reflect the evolutionary relationships between the species. Figure 3 shows the sequence similarity heatmap of myoglobins of different species.

The myoglobin sequences of chicken and turkey were identical, indicating complete sequence conservation among poultry. Additionally, beef and bison myoglobins have identical sequences. Fish species such as yellowfin tuna and tilapia exhibited significantly lower sequence similarity with others, ranging from approximately 41% to 46%. Interestingly, tuna and tilapia shared around 80% similarity, which suggests a moderate level of conservation within the fish species. These results highlight a strong correlation between sequence similarity and the evolutionary history of the species. There is also a significant difference in the primary structure of myoglobins from aquatic, poultry, and livestock.

### 3.2. Structure Validation and Quality Assessment of Myoglobin Modelled Protein

The reliability of homology models generated by SWISS-MODEL is important for subsequent functional analysis and prediction. The structure validation and quality assessment were performed using established metrics to ensure that the predicted structure’s properties are comparable to those of reference structures. The quality of the modeled protein structures was validated through a comparative analysis with reference structures, and the results are summarized in Table 2. The models demonstrated exceptional local accuracy, as indicated by LDDT scores ranging from 0.88 to 0.94. These values, which are close to 1, confirm that the models possess a highly similar fold and backbone to the reference structures. Furthermore, TM-scores (Template Modeling Score) of 0.98 and 0.99 confirmed a near-perfect topological similarity, providing strong support for the overall reliability of the modeled structures. Additional validation metrics reinforced these findings. The MolProbity score was less than 1, signifying a high-quality structure. The low RMSD (Root Mean Square Deviation) values, all below 1 Å, demonstrated minimal deviation from the reference structures at the atomic level. Finally, QMEAN (Qualitative Model Energy Analysis) scores approaching 1 further validate the high quality of the models. Collectively, these results support the reliability of the computationally predicted protein structures.

**Figure 4:**
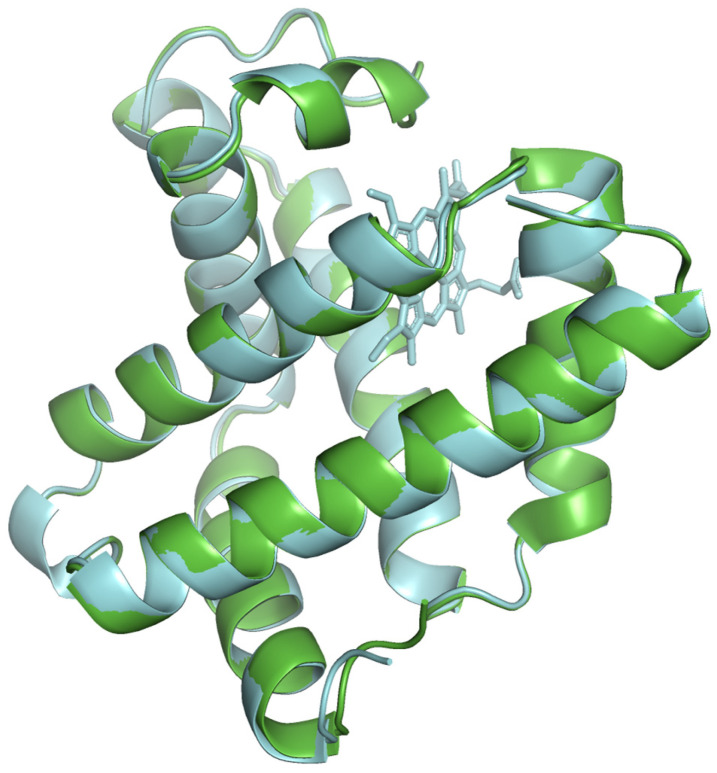
The modeled beef myoglobin structure (cyan color) superimposed with AlphaFold predicted structure AF-P86873 (green color).

**Figure 5:**
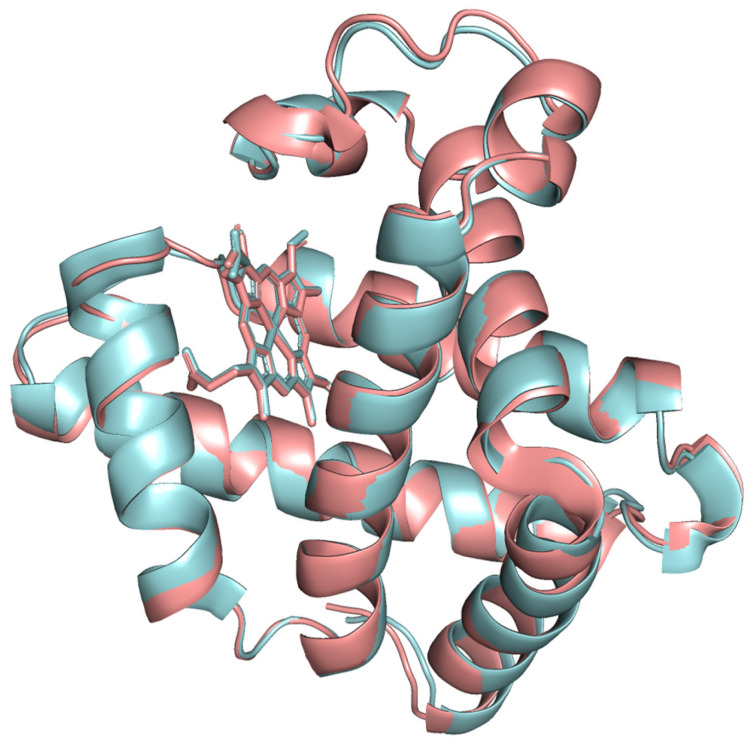
The modeled beef myoglobin structure (cyan color) superimposed with 1MWD X-ray crystal structure (red color).

**Figure 6:**
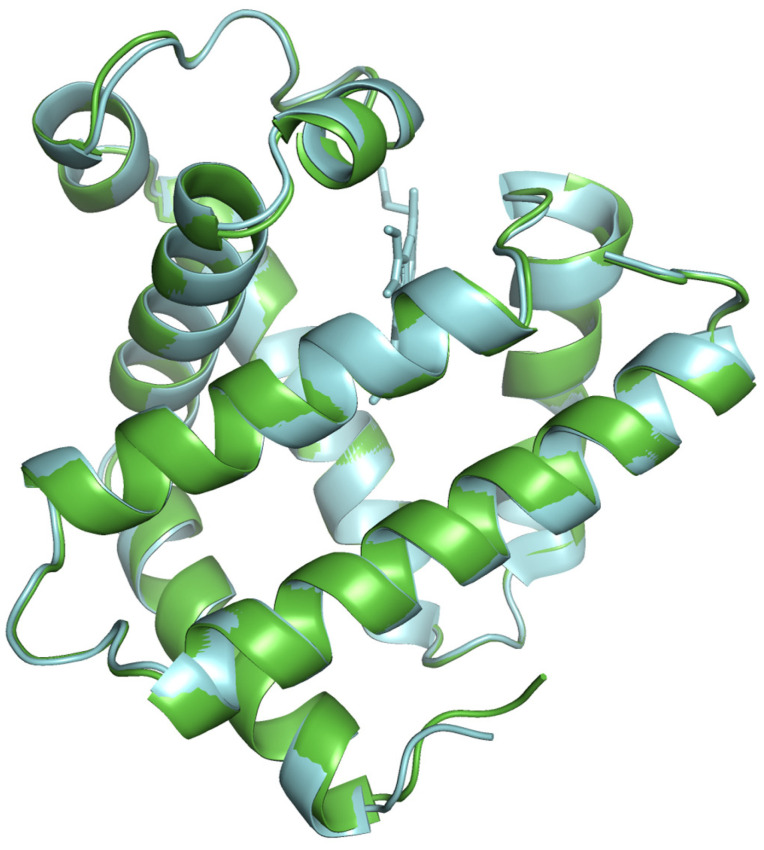
The modeled pork myoglobin structure (cyan color) superimposed with AlphaFold predicted structure AF-P02189 (green color).

The modeled protein structures were superimposed with reference structures to visually assess how the predicted model aligns with reference structures, comparing their overall fold and local features. The superimposed structures are depicted in Figure 4, Figure 5 and Figure 6. The modeled structure superimposes well with the reference structures, demonstrating a close structural match and confirming the structural reliability.

### 3.3. Ligand Accessibility and Myoglobin Oxidation

**Figure 7:**
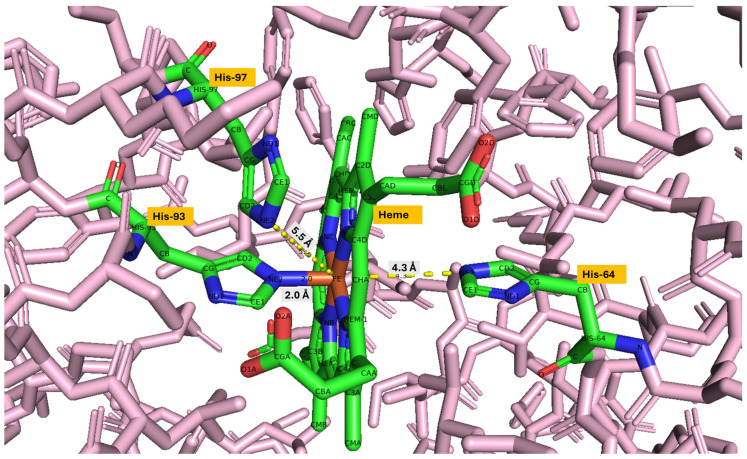
Distance between heme iron and key histidine residues (His-64, His-93, and His-97) for modeled beef myoglobin visualized in PyMOL (Stick model representation). Distances are shown in Å (angstrom) units and measured from iron to NE2 [nitrogen on the ε (epsilon) carbon, position 2 in histidine ring]. The backbone of the beef myoglobin structure is shown in pink sticks, while heme and the residues His-64, His-93, and His-97 are highlighted in a combination of green (carbon), red (oxygen), blue (nitrogen), and brown (iron) color sticks.

The special arrangement between the iron center of the heme and the histidine residues, particularly the proximal and distal histidine, plays a critical role in determining ligand-binding stability and susceptibility to oxidation. In most mammals and birds, the proximal histidine is His93, and the distal histidine is His64. However, in fish myoglobins, these correspond to His88 (proximal) and His59 (distal), reflecting a shift in sequence numbering. Despite this variation, the functional roles of these residues in stabilizing ligand interactions and influencing redox behavior remain conserved across species. Table 3 presents the distance between key histidine residues and the iron (Fe) atom in the heme group of myoglobin across various species, measured from Fe to NE2 (nitrogen on the ε (epsilon) carbon, position 2 in the histidine ring). Figure 7 shows the distance between heme Fe and key histidine residues (His-64, His-93, and His-97) for beef myoglobin visualized in PyMOL.

The distal histidine-Fe distance (His64–Fe/His59–Fe *) is 4.3 Å in mammals and fish, indicating a non-covalent interaction where the distal histidine helps stabilize bound ligands through hydrogen bonding. This distance is slightly longer in chicken and turkey (5 Å), which may imply weaker stabilization of bound ligands. The ~2.0 Å distance between proximal histidine to Fe (His93–Fe/His88–Fe) is well conserved across all species, which is a direct coordination bond between the distal histidine and iron. The His97–Fe/His92–Fe * is not directly bonded to Fe; this residue lies close to the heme pocket and may affect the geometry or electrostatic environment around the heme.

### 3.4. Structural Features of Myoglobin Binding Cavities

**Figure 8:**
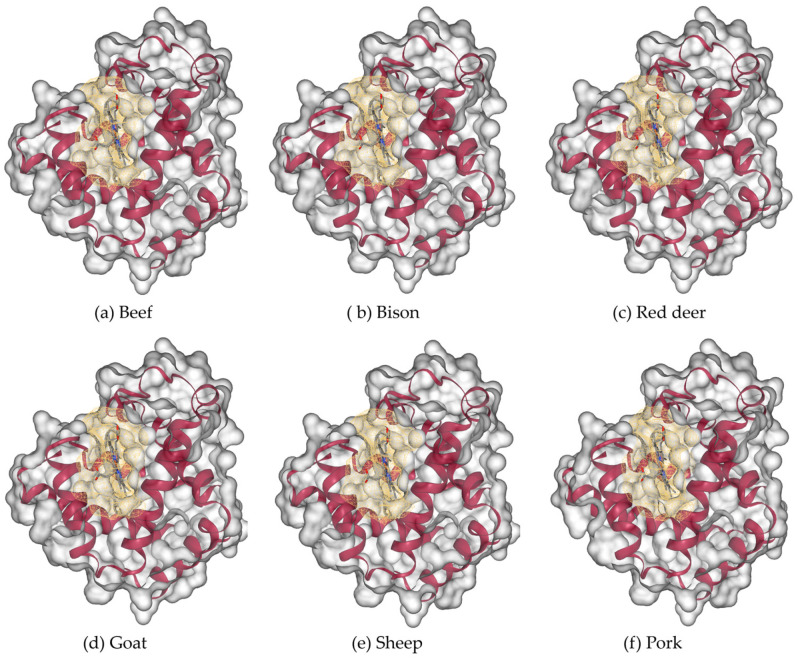
Three-dimensional modeled structure of myoglobin from livestock: (**a**) beef, (**b**) bison, (**c**) red deer, (**d**) goat, (**e**) sheep, and (**f**) pork. Molecular surface (gray color) with the binding cavity depicted in yellow color mesh and alpha helices in red color cartoon forms.

**Figure 9:**
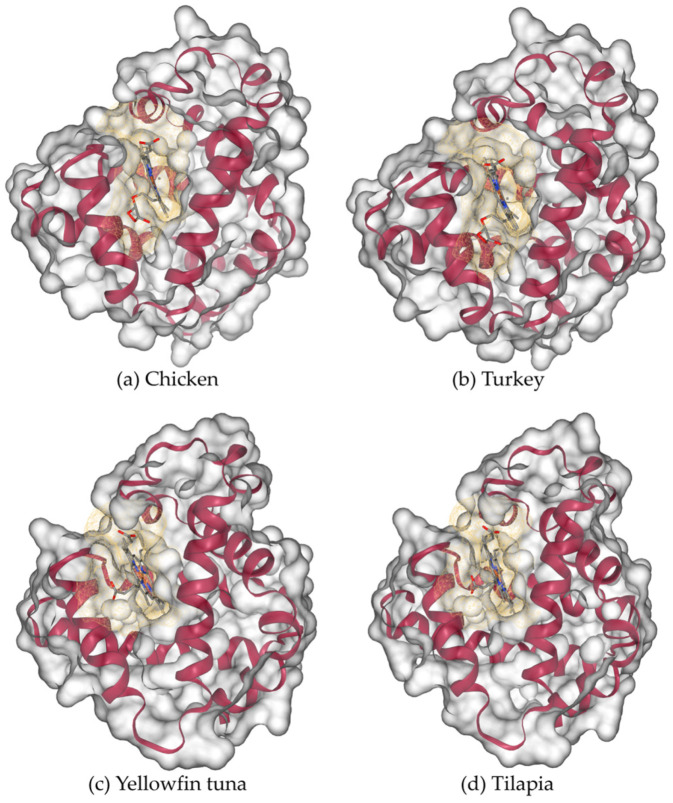
Three-dimensional modelled structure of myoglobin from poultry and fish: (**a**) chicken, (**b**) turkey, (**c**) yellowfin tuna, and (**d**) tilapia. Molecular surface (gray color) with the binding cavity depicted in yellow color mesh and alpha helices in red color cartoon form.

The three-dimensional modelled structure of myoglobin from livestock is shown in Figure 8. Poultry and fish, along with the binding cavities, are shown in Figure 9. Myoglobin’s characteristic α-helical fold (represented by magenta ribbons) forms a compact, globular structure that encloses the central heme prosthetic group. The α-helices fold to create a compact globular structure, within which a binding cavity (shown as a yellow mesh surface) accommodates the heme group. This cavity is defined by surrounding residues: predominantly hydrophobic side chains that stabilize the heme, along with key polar residues (such as the distal and proximal histidines) that facilitate the reversible binding of oxygen to the iron atom.

The number of oxidation-prone residues in myoglobin protein across different species is shown in Table 4.

The data suggests that the mammalian myoglobin is highly conserved in the number of oxidation-prone residues. All of them have 2 tryptophan, 3 methionine, and 2 tyrosine residues. The primary difference is in the number of histidines, with ruminants (beef, bison, sheep, goat, and red deer) having 12 or 13, while pork has 9. Poultry myoglobin contains four methionine residues, in contrast to the three in mammalian myoglobin, which may render it more susceptible to oxidative reactions. This structural difference aligns with the typically shorter shelf-life and more rapid discoloration observed in poultry meat compared to red meat. Fish myoglobins (yellowfin tuna and tilapia) exhibit distinct structural features compared to mammalian and poultry counterparts. Notably, yellowfin tuna myoglobin contains a single cysteine residue, which is absent in other species. Given that cysteine is highly prone to oxidative modification, its presence strongly suggests an increased susceptibility to oxidation, consistent with the rapid discoloration and limited shelf-life characteristic of fish meat. In addition, fish myoglobins possess fewer histidine residues (six in total) and reduced numbers of tryptophan residues compared to mammals and poultry, further highlighting their unique structural composition and potential influence on oxidative stability.

**Figure 10:**
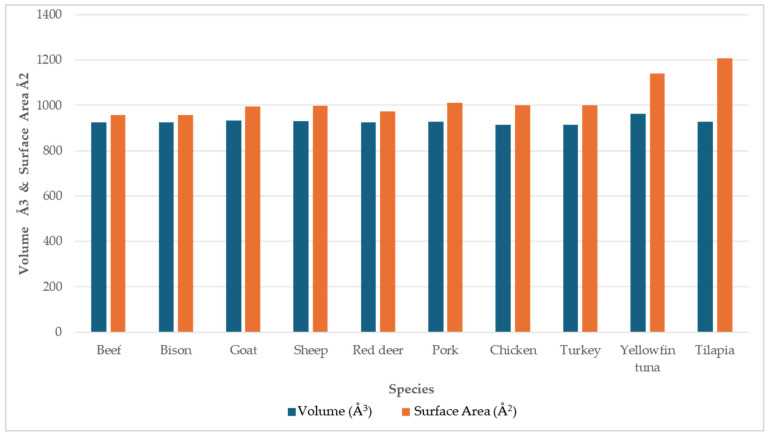
The comparison of pocket cavity volume (Å^3^, in blue) and pocket surface area (Å^2^, in orange) of the protein binding cavity of modelled myoglobin of livestock (beef, bison, sheep, goat, red deer, and pork), poultry (chicken and turkey) and fish (yellow fin tuna and tilapia) from Proteins Plus DoGSite3 binding site detection. Volume represented in Å^3^ (cubic angstrom) unit, and surface area in Å^2^ (square angstrom) unit.

The comparison of the calculated pocket cavity volume (space around heme) and pocket surface area (internal surface of cavity) for myoglobin from ten species is shown in Figure 10. Mammalian and poultry species exhibit relatively uniform cavity metrics, whereas fish, such as myoglobins in tilapia and yellowfin tuna, display larger cavity volumes and surface areas. The heme pocket cavity compactness metric is shown in Table 5. The compactness, calculated as the ratio of the pocket cavity volume to the pocket surface area, reflects the overall shape and tightness of the binding pocket. Higher values indicate a more spherical and tightly packed cavity, whereas lower values correspond to irregular, less compact cavities. Mammalian and poultry species exhibit high compactness values ranging from 0.91 to 0.97, indicating a tight, well-defined cavity. Fish species exhibit low compactness values (yellowfin tuna, 0.84; tilapia, 0.77), which indicates the binding pockets in fish species are more open and irregular. These variations in cavity size, volume, and topology between species may influence heme accessibility, oxygen affinity, and susceptibility to oxidation.

It was observed that there are differences in the α-helical structure of the myoglobin model across different species, as shown in Table 6. The D-helix was observed to be short in a few species within the ruminant and poultry categories. In contrast, it was absent in the fish category.

### 3.5. Solvent-Accessible Surface Area (SASA) of Myoglobin Heme Region in Different Species

Table 7 presents the heme solvent-accessible surface area and buried surface area for all species. The solvent-accessible surface area (SASA) reflects the portion of the heme exposed to solvent, while the buried surface area (BSA) represents the extent of heme shielding by surrounding protein residues. Together, these values provide insights into the degree of heme enclosure and potential susceptibility to oxidation.

Poultry species exhibited the highest SASA values (835.14 Å^2^ for both chicken and turkey) and intermediate buried surface area (~675 Å^2^), indicating greater exposure of the heme to solvent molecules. Myoglobins from livestock species showed the lowest SASA values (819.3 Å^2^ for beef, bison, sheep, goat, red deer, and pork) and buried surface area of ~667 Å^2^. This may contribute to increased oxidative stability of red meat, which in turn offers structural protection against reactive oxygen species. Fish myoglobins’ SASA values of 819.93 Å^2^ and buried surface area ~685–689 Å^2^ for both yellowfin tuna and tilapia reflect partial heme exposure. This indicates the relationship between heme exposure to solvent and oxidative stability across species. Structural differences in solvent accessibility likely contribute to the diverse color and shelf-life stability of different species.

**Figure 11:**
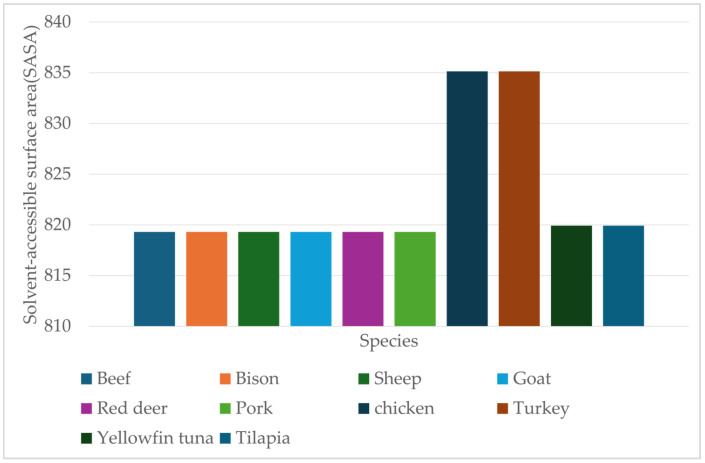
Comparison of heme solvent-accessible surface area (Å^2^) of modelled myoglobin protein of livestock (beef and pork), poultry (chicken and turkey), and fish (yellow fin tuna and tilapia), calculated using PDBePISA. Å^2^ denotes the surface area in square angstroms.

Figure 11 shows the SASA values of heme calculated for ten species representing three categories: livestock (beef, bison, sheep, goat, and red deer), pork, poultry (chicken and turkey), and fish (yellowfin tuna and tilapia).

## 4. Discussion

A quantitative assessment of sequence similarity among species-specific myoglobins was performed using a percentage identity matrix to understand why poultry and fish oxidize more rapidly compared to other species. The analysis revealed pairwise sequence identities ranging from 40% to 88%, reflecting varying degrees of conservation across different vertebrate lineages. This matrix calculates the proportion of identical amino acids at aligned positions between sequences, offering insights into both evolutionary relationships and potential functional similarities. High sequence similarity typically correlates with structural conservation, as homologous proteins often preserve similar three-dimensional folds [27]. Thus, sequence similarity serves as a valuable indicator for inferring structural and functional homology among myoglobins from diverse species.

Compared to other species, the presence of cysteine residues in fish myoglobin is considered an evolutionary adaptation to oxidative stress and environmental variability in aquatic habitats. Unlike terrestrial mammals, which typically lack cysteine in myoglobin, many fish species possess non-conserved cysteine residues that serve protective or regulatory functions. The thiol group (-SH) of cysteine can act as a redox-active center, helping to scavenge reactive oxygen species generated during hypoxia–reoxygenation cycles common in aquatic environments [28]. Additionally, cysteine can undergo reversible S-nitrosylation, potentially modulating nitric oxide signaling and vascular regulation in fish tissues [29]. In some species, cysteine may form intra- or intermolecular disulfide bonds under oxidative conditions, contributing to protein stability, especially in cold or high-pressure environments such as those inhabited by Antarctic notothenioid fish [30,31]. Furthermore, cysteine residues may assist in metal ion binding or detoxification in polluted waters. For example, the single cysteine found in tuna myoglobin is hypothesized to reduce the rate of metmyoglobin formation, thereby maintaining functional oxygen transport in this highly active species [32]. These adaptations highlight the role of cysteine in enhancing myoglobin function and resilience under diverse aquatic conditions.

Secondary structural elements are critical for establishing the functional three-dimensional architecture of proteins. In all species examined, myoglobin exhibited a predominant α-helical conformation, consistent with the well-characterized globin fold.

In addition to well-established oxidation-prone residues such as cysteine and methionine, several other amino acids—including histidine, proline, arginine, lysine, threonine, tyrosine, and tryptophan—are susceptible to oxidative modifications, especially under stress conditions such as changes in pH or temperature [33]. A quantitative assessment of oxidation-prone sites across species revealed that pork myoglobin has approximately 24 susceptible sites, whereas chicken and turkey each exhibit up to 30 sites, coinciding with their elevated disorder content and greater solvent accessibility.

Protein oxidation, primarily driven by reactive oxygen species (ROS) and lipid peroxidation byproducts, can lead to structural degradation, particularly in the side chains and backbone of sensitive amino acids. Such modifications have been directly linked to the deterioration of meat quality attributes, including color, tenderness, and flavor [34]. These findings underscore the role of secondary structure and amino acid composition in modulating the oxidative stability of species-specific myoglobin.

The spatial arrangement of key histidine residues—proximal (His93) and distal (His64)—plays a critical role in myoglobin’s ability to bind and stabilize oxygen. Comparative analysis of the modeled three-dimensional structures revealed subtle interspecies differences in the distance between the histidine and the heme iron, which may influence the oxygen-binding capacity of myoglobin across ruminants, poultry, and fish. The proximal histidine coordinates directly with the heme iron, and its effect is influenced by spatial proximity, electrostatic environment, and atomic positioning. In contrast, distal effects involve the distal histidine and surrounding pocket residues (e.g., tyrosine, glutamine) that form hydrogen bonds with bound oxygen, contributing to ligand stabilization. The size, shape, polarity, and charge distribution within the distal pocket critically influence the efficiency of oxygen binding [35].

To further explore structural variation, binding cavity characterization was performed using pocket detection tools. Differences in heme cavity volume and surface area among species were quantified to assess how these parameters may modulate the accessibility of oxidizing agents to the heme group. Larger or more accessible cavities may allow greater interaction with reactive oxygen species, contributing to increased susceptibility to oxidation.

Additionally, analysis of solvent-accessible surface area (SASA) near the heme revealed interspecies variation, with higher accessibility corresponding to enhanced oxidative vulnerability. These results support the idea that protein cavities—including binding pockets, internal voids, and access tunnels—collectively define the topological features of myoglobin, which in turn affect ligand pathway dynamics and oxidative behavior. The combined assessment of cavity geometry and histidine coordination provides valuable insight into structure-function relationships across species-specific myoglobins. Previous research also reported the absence of D-helix in fish species [36].

Role of proteoforms in species-specific oxidation: In postmortem muscle, studies have shown that proteoforms, especially post-translational modifications, can influence myoglobin oxidation. However, current knowledge on post-translational modification is limited regarding all ten species of myoglobin considered in this research. Research on myoglobin from aged beef longissimus muscles noted phosphorylation and HNE alkylation at various amino acids [37] and suggested its potential role in meat discoloration. Future studies are needed to better understand the role of proteoforms in species-specific meat discoloration.

## 5. Conclusions

Although evolutionary and physiological adaptations influence the functions of myoglobin in different species, this research has noted structural differences in poultry and aquatic species that affect myoglobin oxidation postmortem. The multiple sequence alignment of the primary sequence demonstrated the evolutionary relationship among ruminants, non-ruminants, poultry, and fish myoglobin. Tuna myoglobin has a cysteine residue, and both fish species have fewer amino acids compared with other species. The secondary structural analysis from different species demonstrated the predominant alpha helical state. Analysis of predicted three-dimensional myoglobin protein models explained the interactions between the heme porphyrin ring and the surrounding amino acid residues, including the proximal and distal histidine, which have a significant role in maintaining structural stability. Poultry has more solvent-accessible surface area compared with other species, while fish species have no D-helix. The heme cavity area is greater in fish species than in different livestock species. The current results suggest that structural differences can increase oxidation in poultry and fish myoglobins compared to myoglobins from livestock species. Understanding the fundamental differences in myoglobin oxidation can be helpful in designing packaging or antioxidant addition strategies to minimize losses due to changes in meat color.

## Figures and Tables

**Table 1:** Amino acid sequences from different categories of species.

Category	Species	Sequence	
Ruminants	Beef	GLSDGEWQLVLNAWGKVEADVAGHGQEVLIRLFTGHPETLEKFDKFKHLKTEAEMKASED LKKHGNTVLTALGGILKKKGHHEAEVKHLAESHANKHKIPVKYLEFISDAIIHVLHAKHP SDFGADAQAAMSKALELFRNDMAAQYKVLGFHG	60 120 153
	Bison	GLSDGEWQLVLNAWGKVEADVAGHGQEVLIRLFTGHPETLEKFDKFKHLKTEAEMKASED LKKHGNTVLTALGGILKKKGHHEAEVKHLAESHANKHKIPVKYLEFISDAIIHVLHAKHP SDFGADAQAAMSKALELFRNDMAAQYKVLGFHG	60 120 153
	Goat	GLSDGEWTLVLNAWGKVEADVAGHGQEVLIRLFTGHPETLEKFDKFKHLKTGAEMKASED LKKHGNTVLTALGGILKKKGHHEAEVKHLAESHANKHKIPVKYLEFISDAIIHVLHAKHP SDFGADAQGAMSKALELFRNDMAAQYKVLGFQG	60 120 153
	Sheep	GLSDGEWQLVLNAWGKVEADVAGHGQEVLIRLFTGHPETLEKFDKFKHLKTEAEMKASED LKKHGNTVLTALGGILKKKGHHEAEVKHLAESHANKHKIPVKYLEFISDAIIHVLHAKHP SDFGADAQGAMSKALELFRNDMAAQYKVLGFQG	60 120 153
	Red deer	GLSDGEWQLVLNAWGKVEADVAGHGQEVLIRLFTGHPETLEKFDKFKHLKTEAEMKASED LKKHGNTVLTALGGILKKKGHHEAEVKHLAESHANKHKIPVKYLEFISDAIIHVLHAKHP SNFGADAQGAMSKALELFRNDMAAQYKVLGFQG	60 120 153
Non-Ruminant	Pork	GLSDGEWQLVLNVWGKVEADVAGHGQEVLIRLFKGHPETLEKFDKFKHLKSEDEMKASED LKKHGNTVLTALGGILKKKGHHEAELTPLAQSHATKHKIPVKYLEFISEAIIQVLQSKHP GDFGADAQGAMSKALELFRNDMAAKYKELGFQG	60 120 153
Poultry	Chicken	GLSDQEWQQVLTIWGKVEADIAGHGHEVLMRLFHDHPETLDRFDKFKGLKTPDQMKGSED LKKHGATVLTQLGKILKQKGNHESELKPLAQTHATKHKIPVKYLEFISEVIIKVIAEKHA ADFGADSQAAMKKALELFRNDMASKYKEFGFQG	60 120 153
	Turkey	GLSDQEWQQVLTIWGKVEADIAGHGHEVLMRLFHDHPETLDRFDKFKGLKTPDQMKGSED LKKHGATVLTQLGKILKQKGNHESELKPLAQTHATKHKIPVKYLEFISEVIIKVIAEKHA ADFGADSQAAMKKALELFRNDMASKYKEFGFQG	60 120 153
Fish	Yellowfin tuna	ADFDAVLKCWGPVEADYTTMGGLVLTRLFKEHPETQKLFPKFAGIAQADIAGNAAISAHG ATVLKKLGELLKAKGSHAAILKPLANSHATKHKIPINNFKLISEVLVKVMHEKAGLDAG GQTALRNVMGIIIADLEANYKELGFSG	60 120 146
	Tilapia	GDFDAVLKHWGPVEADYTGYGSLVLTRLFTEHPETQKLFPKFVGIPQGELASSSAVADHG ATVLKKLGELLKAKGNHAAILKPLANSHATKHKIPINNFKLISEVIVKVFAEKAGLDTAG QQGLRNVMSKVIADLEASYKELGFTG	60 120 146

Note: The N-terminal methionine was removed from the sequence before modeling to represent the biologically mature form; therefore, it is not shown in the sequences above.

**Table 2:** The comparative scores of beef and pork myoglobin protein structure models generated by SWISS-MODEL (models in the present study) with experimental and AlphaFold structures.

SWISS-MODEL	Reference Structure	LDDT	TM-Score	RMSD	MolProbity Score	QMEAN
Beef	1MWD (Sus scrofa) 153aa X-ray crystal structure	0.88	0.98	0.73	0.75	0.06
	AF-P86873-F1 (Bos bison) 154aa AlphaFold structure	0.93	0.99	0.39	0.75	0.06
Pork	1MWD (Sus scrofa) 153aa X-ray crystal structure	0.91	0.98	0.73	0.64	0.02
	AF-P02189-F1 (Bos bison) 154aa AlphaFold structure	0.94	0.99	0.35	0.64	0.02

**Table 3:** The distances (in angstrom; Å) between histidine residues and the heme Fe atom in modelled myoglobin structures across different species.

Residue	Beef	Bison	Sheep	Goat	Red Deer	Pork	Turkey	Chicken	Yellowfin Tuna	Tilapia
His64 Nε–Fe/ His59 Nε–Fe *	4.3	4.3	4.3	4.3	4.3	4.3	5.0	5.0	4.3	4.3
His93 Nε–Fe/ His88 Nε–Fe *	2.0	2.0	2.0	2.0	2.0	2.0	2.1	2.1	2.0	2.0
His97 Nε–Fe/ His92 Nε–Fe *	5.5	5.5	5.5	5.5	5.5	5.5	6.0	6.0	5.6	5.6

Note: * The histidine residues for fish [His88 (proximal) and His59 (distal)] are different from those of other species.

**Table 4:** Number of each type of oxidation-prone residues in myoglobin protein across all species.

Species	Cysteine (C)	Tryptophan (W)	Methionine (M)	Tyrosine (Y)	Histidine (H)	Total Number
Beef		2	3	2	13	20
Bison		2	3	2	13	20
Sheep		2	3	2	12	19
Goat		2	3	2	12	19
Red deer		2	3	2	12	19
Pork		2	3	2	9	16
Chicken		2	4	2	9	17
Turkey		2	4	2	9	17
Yellowfin	1	1	2	2	6	12
Tilapia	*	1	1	3	6	11

* Tilapia (UniProt number: I3KNL0) myoglobin does not contain a cysteine residue.

**Table 5:** Heme cavity compactness across all species.

Species	Compactness
Beef	0.97
Bison	0.97
Goat	0.94
Sheep	0.93
Red deer	0.95
Pork	0.92
Chicken	0.91
Turkey	0.91
Yellowfin tuna	0.84
Tilapia	0.77

**Table 6:** Differences in occurrences of D-helix in modelled myoglobin across different species.

Species	Occurrence	Length in Residues	Remarks
Beef	Present	19	Present
Bison	Present	19	Present
Sheep	Present	5	Short helix present
Goat	Present	5	Short helix present
Red deer	Present	5	Short helix present
Pork	Present	5	Short helix present
Chicken	Present	5	Short helix present
Turkey	Present	5	Short helix present
Yellowfin tuna	Absent		
Tilapia	Absent		

**Table 7:** Solvent-accessible and buried surface areas of the heme group in myoglobin from different species.

Species	Heme—Solvent-Accessible Surface Area (Å^2^; SASA)	Heme—Buried Surface Area (Å^2^; BSA)
Beef	819.3	666.69
Bison	819.3	666.69
Sheep	819.3	666.69
Goat	819.3	666.69
Red deer	819.3	667.18
Pork	819.3	667.24
Chicken/Turkey	835.14	675.22
Yellowfin tuna	819.93	685.52
Tilapia	819.93	689.34

Note: Å^2^ denotes the surface area in square angstroms.

## Data Availability

The data will be available upon request to the corresponding authors.
